# Two Cases of Recessive Intellectual Disability Caused by *NDST1* and *METTL23* Variants

**DOI:** 10.3390/genes11091021

**Published:** 2020-08-31

**Authors:** Amjad Khan, Zhichao Miao, Muhammad Umair, Amir Ullah, Mohammad A. Alshabeeb, Muhammad Bilal, Farooq Ahmad, Gudrun A. Rappold, Muhammad Ansar, Raphael Carapito

**Affiliations:** 1 Laboratoire d’ImmunoRhumatologie Moléculaire, Plateforme GENOMAX, INSERM UMR_S 1109, Faculté de Médecine, Fédération Hospitalo-Universitaire OMICARE, Fédération de Médecine Translationnelle de Strasbourg (FMTS), LabEx *TRANSPLANTEX*, Université de Strasbourg, 67085 Strasbourg, France; amjadkhanqau123@hotmail.com; 2 European Molecular Biology Laboratory, European Bioinformatics Institute (EMBL-EBI), Wellcome Genome Campus, Cambridge CB10 1SD, UK; chichaumiau@gmail.com; 3 Shanghai Fourth People’s Hospital Affiliated to Tongji University School of Medicine, No.1878 North Sichuan Road, Hongkou District, Shanghai 200081, China; 4 Medical Genomics Research Department, King Abdullah International Research Center (KAIMRC), King Saud Bin Abdulaziz University for Health Sciences, King Abdulaziz Medical City, Ministry of National Guard Health Affairs, Riyadh 11481, Saudi Arabia; khugoo4u@yahoo.com; 5 Nephrology and Dialysis Unit, District Head Quarter Teaching Hospital, Bannu 28100, Pakistan; amirullah.amc@gmail.com; 6 Developmental Medicine Department, King Abdullah International Medical Research Center (KAIMRC), King Saud Bin Abdulaziz University for Health Sciences, Ministry of National Guard-Health Affairs (MNGHA), Riyadh 11481, Saudi Arabia; shabeebonline@hotmail.com; 7 Department of Biochemistry, Faculty of Biological Sciences, Quaid-i-Azam University, Islamabad 45320, Pakistan; bilalmuhammadarain@gmail.com (M.B.); mansar_76@yahoo.com (M.A.); 8 Department of Chemistry, Women University Swabi, Khyber Pakhtunkhwa 23430, Pakistan; farooq.amazai@gmail.com; 9 Department of Human Molecular Genetics, Institute of Human Genetics, Ruprecht-Karls-University, 69118 Heidelberg, Germany; gudrun.rappold@med.uni-heidelberg.de; 10 Service d’Immunologie Biologique, Plateau Technique de Biologie, Pôle de Biologie, Nouvel Hôpital Civil, Hôpitaux Universitaires de Strasbourg, 1 Place de l’Hôpital, 67091 Strasbourg, France

**Keywords:** autosomal recessive intellectual disability, *NDST1*, *METTL23*, exome sequencing

## Abstract

Intellectual disability (ID) is a highly heterogeneous genetic condition with more than a thousand genes described so far. By exome sequencing of two consanguineous families presenting hallmark features of ID, we identified two homozygous variants in two genes previously associated with autosomal recessive ID: *NDST1* (c.1966G>A; p.Asp656Asn) and *METTL23* (c.310T>C; p.Phe104Leu). The segregation of the variants was validated by Sanger sequencing in all family members. In silico homology modeling of wild-type and mutated proteins revealed substantial changes in the secondary structure of both proteins, indicating a possible effect on function. The identification and validation of new pathogenic *NDST1* and *METTL23* variants in two cases of autosomal recessive ID further highlight the importance of these genes in proper brain function and development.

## 1. Introduction

Intellectual disability (ID) is a heterogeneous complex brain disorder with an early onset of cognitive impairment, characterized by substantial limitations in intellectual functioning and behavioral adaptations [1]. ID is clinically distinguished in syndromic and non-syndromic forms and the overall incidence varies from 1 to 3% [2,3]. Both environmental and genetic factors have been associated with ID pathogenesis. Environmental factors such as infections, iodine-deficient diet, ototoxic drug treatment, poor nutrition, prenatal/perinatal brain ischemia, prenatal/postnatal infections (meningitis, encephalitis), hypoglycemia, prenatal exposure to alcohol or other teratogens, and inadequate medical services have been described. The genetic causes are diverse and include chromosomal aberrations and single or multiple gene defects [4,5,6]. Single-nucleotide variants (SNV), indels and copy-number variations (CNVs) have been identified as major variant types causing ID [7,8]. ID can be inherited as an autosomal recessive (AR), autosomal dominant (AD), X-linked recessive (XLR), X-linked dominant (XLD) or mitochondrial disease [9]. The phenotypic spectrum of ID is wide and more than a thousand genes have been associated with the condition [7,10,11]. Genetic diagnoses are considered one of the essential parts of medical genetics for the prediction, prevention as well as the prognosis or even treatment of ID.

Here, we present the molecular analysis of two consanguineous Pakistani families by exome sequencing. We identified the pathogenic variants in N-deacetylase and N-sulfotransferase 1 (NDST1) and methyltransferase-like 23 (*METTL23*) genes segregating with the disease in an autosomal recessive manner. *NDST1* encodes a bifunctional GlcNAc N-deacetylase/N-sulfotransferase with important functions in heparan sulfate biosynthesis. *METTL23* is strongly predicted to function as an S-adenosylmethionine (SAM)-dependent methyltransferase that catalyzes the transfer of a methyl group on diverse substrates including nucleic acids, proteins, lipids and small metabolites. Both genes have previously been associated with intellectual disability and development delay.

## 2. Materials and Methods

### 2.1. Study Subjects

In the current study, two consanguineous families, presenting recessive ID from the Khyber Pakhtunkhwa Province of Pakistan were recruited. Clinical information including age, gender, family history and consanguinity was recorded. Blood samples from both family members were obtained after informed consent was signed by all included members.

### 2.2. DNA Extraction and Quantification

Genomic DNA was extracted from peripheral blood using the QIAquick DNA extraction kit (Qiagen, Hilden, Germany) and quantified using the Nanodrop-2000 spectrophotometer (ThermoFisher Scientific, Waltham, MA, USA).

### 2.3. Library Construction and Exome Sequencing

Library construction was performed with the Ion AmpliSeq Library Kit 2.0 according to the manufacturer’s instructions (ThermoFisher Scientific, Waltham, MA, USA). The libraries were diluted at 100 pmol/L and were subjected to emulsion polymerase chain reaction (emPCR) using the OneTouch 2 instrument with an Ion Proton I Template OT2 200 Kit v3, and the Template-positive Ion Sphere Particles enrichment was achieved by the Ion One Touch enrichment system (ThermoFisher Scientific, Waltham, MA, USA). Ion Proton I chip was prepared and loaded on the Ion Proton sequencer following the manufacturer’s instructions.

### 2.4. Data Processing

The sequence data was aligned to the reference human genome (GRCh37/hg19) using the Ion Torrent Mapping Alignment Program (ITMAP, ThermoFisher Scientific, Waltham, MA, USA). The Torrent Variant Caller (TVC) plugin was used for the genotyping of multi-allelic substitutions and indels (ThermoFisher Scientific, Waltham, MA, USA). Annotation of the variants was performed using ANNOVAR (http://www.wannovar.usc.edu/) and the sequence reads were visualized using the Integrated Genomic Viewer (IGV, http://www.broadinstitute.org/igv/).

### 2.5. Variant Filtration Steps

All the variants were screened according to the location, frequency and type of variation. Variants were filtered with a minor allele frequency (MAF) cutoff of 1% in the Exome Variant Server (http://evs.gs.washington.edu/EVS/), GnomAD (https://gnomad.broadinstitute.org), and 1000 Genomes (http://www.1000genomes.org/). The analysis focused on non-synonymous SNVs (missense, non-sense, splice-site, and frameshift) and was submitted to Polyphen-2 (http://genetics.bwh.harvard.edu/pph2/), Sorting Intolerant from Tolerant (SIFT, http://sift.jcvi.org/), Protein Variation Effect Analyzer (PROVEAN, http://provean.jcvi.org), Mutation Taster (http://www.mutationtaster.org/), Varsome (https://varsome.com/), Mutation assessor (http://mutationassessor.org), and Combined Annotation Dependent Depletion (CADD, https://cadd.gs.washington.edu/) for functional effect prediction. Finally, for the interpretation of variants, the American College of Medical Genetics and Genomics (ACMG) 2015 guidelines were used [12].

### 2.6. Primer Design and Variant Confirmation

Gene Runner (version 5.0.69 Beta, Hastings, NY, USA) software was used for primer design (*NDST1*; Forward primer: 5′-ACCAGAGAAAGGCAGAGACC-3′ Reverse Primer: 5′-GTCACTGCACTTTGTCCCTG-3′, *METTL23*, Forward primer: 5′-TAGATTGGAGCTGGAGTGAG-3′ and Reverse Primer: 5′-GGACCTTGGGATTCTTGTGC-3′) and Sanger sequencing was performed using an ABI3730xl Automated Sequencer to verify the co-segregation of the identified variants with the disease phenotype (Thermo Fisher Scientific, Waltham, MA, USA). The Sanger sequencing results were examined and compared with Chromas Lite (http://technelysium.com.au/wp/) and Codon Code Aligner (https://www.codoncode.com/aligner/).

### 2.7. Homology Protein Modeling

#### 2.7.1. NDST1 (p.Asp656Asn)

The crystal structure of the NDST1 protein was retrieved from the protein data bank (PDB ID: 1NST; 2.3 Å resolution), while 3-O-sulfotransferase (3-OST-1, PDB ID: 3UAN; 1.84 Å resolution) was used to model the dimerization effect of NDST1. 3-O-sulfotransferase shares high structural similarity with NDST1: the two domains of 3-O-sulfotransferase have 0.761 and 0.813 Å Root-Mean-Square Deviation (RMSD) from NDST1. The 1NST structure was superimposed to the two domains of 3UAN to model the dimer structure of NDST1.

#### 2.7.2. METTL23 (p.Phe104Leu)

We used the METTL21C protein (PDB ID: 4MTL, resolution 1.6 Å), which shares a sequence identity of 39.4% with METTL23, as a structure template to model the structure of METTL23. There are other human methyltransferase-like proteins, which have high sequence similarity to METTL23, including METTL21A (PDB ID: 4LEC, resolution 2.3 Å), METTL21B (PDB ID: 4QPN, resolution 1.3 Å), METTL21D (PDB ID: 4LG1, resolution 1.8 Å). All these structures are highly similar in structure, sharing RMSD values within 0.9 Å. This confirms that the structure modeling of METTL23 using these templates is highly reliable. SWISS-MODEL [13] was used to build the 3D model, while the ‘mutagenesis’ plugin in PyMOL (The PyMOL Molecular Graphics System, Version 2.0 Schrödinger, LLC, New York, NY, USA) was used to model the single residue mutation.

## 3. Results

### 3.1. Clinical Manifestations

In this study, we recruited two consanguineous families affected by ID from Pakistan. All the affected individuals were examined by a clinical neurologist at District Headquarters Hospital Bannu.

#### 3.1.1. Family A

The members of family A live in the Bannu districts of Khyber Pakhtunkhwa Province of Pakistan. This four-generation family includes two siblings, one male (IV-1) and one female (IV-2), with cognitive impairment that were born from consanguineous parents. The pregnancy and delivery were unremarkable. The parents of the affected individuals are phenotypically normal and have no neurological symptoms related to ID. Patients IV-1 and IV-2 were examined at the age of 10 and 12 years, respectively. Clinical examination revealed short stature (−2.6SD and −3.9SD for IV-1 and IV-2, respectively), early onset epilepsy and seizures at 2–3 years of age, delayed development, speech delay, substantially limited learning abilities with IQs below 40, and limited communication skills. Dysmorphic features include downward slanting large eyes and eyebrows, depressed nasal bridge, and short upturned nose. Besides ID, the physical assessment revealed behavioral abnormalities (aggression), mild hypotonia, sleep disturbances and developmental delay. No cardiac, respiratory, skeletal or skin anomalies were observed. Their vision and hearing were normal. Additional clinical information of the affected individuals is summarized in Table 1.

#### 3.1.2. Family B

**Figure 1:**
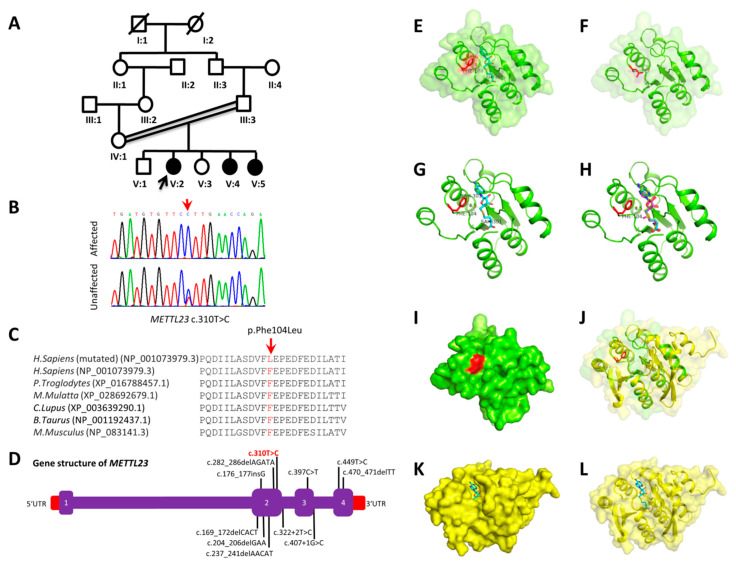
(**A**) Pedigree of family B, exhibiting autosomal recessive inheritance. (**B**) Sanger sequencing results of family (B). The red arrow indicates the localization of the variant. (**C**) Conservation of Phe104 across different species. (**D**) Gene structure of *METTL23*. Known variants are indicated on the gene structure. The c.310T>C variant is shown in red. (**E**) The modeled structure in complex with the SAH ligand, which was superimposed according to the METTL21C structure. (**F**) The Phe104Leu mutation in the modeled structure. (**G**) Same as (**E**) without showing the molecular surface. (**H**) The superimposition of the SAH and SAM ligands from the METTL21A, METTL21B and METTL21C. (**I**) The molecular surface of the modeled structure of METTL23. (**J**) The superimposition between the modeled METTL23 and crystal structure of METTL21C. (**K**) The molecular surface of METTL21C. (**L**) The structure and molecular surface of METTL21C.

Family B also originates from the Khyber Pakhtunkhwa province of Pakistan and the parents are second cousins (Figure 1A). The family comprises three affected sisters (V-2, V-4, and V-5) out of five siblings, indicating an autosomal recessive inheritance. All three patients had cognitive defect and their estimated IQ values fall in the range of severe ID (20–25 to 35–40). Epilepsy and seizures were present at 1.5–2 years of age. Last clinical examination, at 10, 12 and 24 years of age for V-2, V-4 and V-5, respectively, revealed ID, speech impairment, developmental delay, behavioral abnormalities (aggression), and mild hypotonia. Dysmorphic features include prominent large-size eyes, eyebrows, ears, short upturned nose with flat nasal bridge, and thin upper lip. Prenatal, perinatal and neonatal medical records of all patients were normal. Additional clinical information of the affected individuals is summarized in Table 1.

### 3.2. Molecular Evaluation

**Figure 2:**
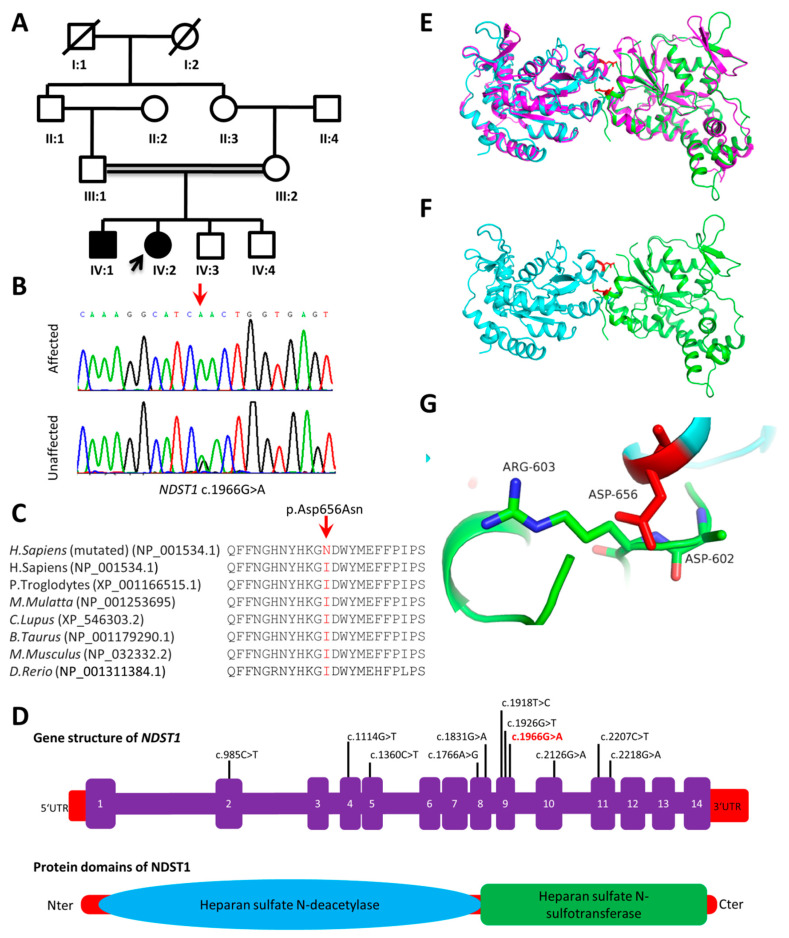
(**A**) Pedigree of family A, exhibiting an autosomal recessive inheritance. Males are represented by squares, females by circles. Filled and unfilled symbols illustrate the affected and unaffected individuals, respectively. A diagonal line over a circle or square represents deceased individuals. Consanguinity is represented by double lines (=). (**B**) Sanger sequencing results of family A. The red arrow indicates the localization of the variant. (**C**) Conservation of Asp656 across different species. The sequence shown is part of the heparin sulfate N-sulfotransferase domain. (**D**) Gene structure and protein domains of NDST1. Known variants are indicated on the gene structure. The c.1966G>A variant is shown in red. (**E**) Modeling the NSDT1 dimer structure using 3-O-sulfotransferase as a template (colored as magenta). Two copies of NSDT1 (green and cyan) are superimposed to the two domains of 3-O-sulfotransferase. The Asp656Asn mutation site is shown as a red stick model. (**F**) The dimer structure of NSDT1 without the 3-O-sulfotransferase template. The Asp656Asn mutation lies on the binding interface. (**G**) The proximal interaction between Asp656Asn and the Asp602 and Arg603 residues on the other interacting domain.

By the exome sequencing of the probands (IV-2 in family A and V-2 in family B), we identified two homozygous missense variants: one in *NDST1* (c.1966G>A; p.Asp656Asn) in family A (Figure 2B) and one in *METTL23* (c.310T>C; p.Phe104Leu) in family B (Figure 1B). In both families, in addition to the patients, both parents and all the non-affected siblings were Sanger sequenced for segregation analysis. The identified variants in *NDST1* and *METTL23* segregated with the disease phenotype in a recessive manner (Figure 1B and Figure 2B) and none of them were present in 200 ethnically matched control individuals. The *METTL23* variant (c.310T>C; p.Phe104Leu) has not been reported in public databases whereas the *NDST1* variant (c.1966G>A; p.Asp656Asn) has a frequency of 0.000016 in the GnomAD dataset, but the homozygous form of the variant was found to be absent. Both variants were highly conserved across various species (Figure 1C and Figure 2C). Online mutation analysis tools predicted both variants as damaging and disease causing (Table 2).

### 3.3. Homology Modeling

#### 3.3.1. NDST1 (p.Asp656Asn)

We modeled the dimer structure of the NDST1 protein using the crystal structures of the NDST1 and 3-O-sulfotransferase proteins. The Asp656Asn mutation lies in the sulfotransferase domain (Figure 2E), directly at the interface of the dimer structure and may have proximal contacts with the Asp 602 and Arg 603 residues. The Asp656Asn mutation changes the electrostatics property of the dimerization interface, and thus may affect the forming of the functional dimer structure which is important for the enzymatic activity of the protein (Figure 2E–G).

#### 3.3.2. METTL23 (p.Phe104Leu)

According to the known crystal structures of METTL21A, METTL21B, METTL21C and METTL21D, the human methyltransferase-like proteins have a S-Adenosyl-L-homocysteine (SAH) or S-adenosylmethionine (SAM) binding pocket. The METTL23 protein shares high sequence similarity with these protein structures. It is highly possible that METTL23 also needs to bind to SAH/SAM to carry out its function. Through structure superimposition between the modeled structure and the known SAH binding complex of METTL21C, the Phe104Leu mutation is close to (4.5 Å from) the SAH ligand. The Phe104 residue may either form pi–pi interaction with the SAH/SAM ligand or form the path to the ligand binding pocket. Therefore, the Phe104Leu mutation may affect the ligand binding property of the protein and impair the protein function (Figure 1E–L).

## 4. Discussion

ID is a heterogeneous disorder with more than a thousand genes identified to date [7,10,11]. Consanguineous families represent a rich resource for the identification of novel disease genes and variants [14]. Here, we examined two consanguineous families with ID segregating in an AR manner.

Using exome sequencing, we identified variants in *NDST1* and *METTL23* genes previously described to cause similar ID phenotypes [15,16]. These genes are highly expressed in the brain and play an important role in synaptic plasticity and dendritic spine morphogenesis [15,16,17]. Both *NDST1* and *METTL23* genes have been associated with neurodevelopmental disorders in corresponding knockout animal models [15,16].

In family A, the patients carrying the homozygous variant in *NDST1* had moderate ID, aggressive behavior, mild delay in ambulation and early stage epilepsy. The *NDST1* gene (NM_001543.5) is composed of 14 exons encoding an 882 amino acids protein (NP_001534.1). It is a member of the heparan sulfate/heparin GlcNAc N-deacetylase/N-sulfotransferase family and a type II transmembrane protein existing in Golgi bodies. It catalyzes the transfer of sulfate from 3′-phosphoadenosine 5′-phosphosulfate to the nitrogen of glucosamine in heparan sulfate. Reuter et al. reported eight patients having ID, hypotonia, ataxia, seizures, and short stature with homozygous missense *NDST1* mutations located within the sulfotransferase domain [15]. The variant identified in family A also resides in the sulfotransferase domain and is associated with features including ID, short stature, aggressive behavior and global developmental delay. Knockdown of the orthologous *Ndst1b* gene in zebrafish causes developmental delay, shortened body and abnormal craniofacial cartilage [18]. Mutant mice with a disruption of *Ndst1* show severe developmental defects including cerebral hypoplasia, lack of olfactory bulbs, eye defects and axon guidance errors [19]. To date, only seven disease-causing variants in *NDST1* have been associated with AR ID (Supplementary Table S1).

Affected individuals in family B carry a novel homozygous missense variant (c.310T>C; p.Phe104Leu) in *METTL23*. The *METTL23* gene (NM_001206984.3) encodes a 190 amino acid-long transcriptional regulator (NP_001073979.3), which is a partner of the alpha subunit of the GA-binding protein transcription factor [17]. Mutations in this gene have been associated with mild recessive ID. The *METTL23* variant identified in our patients shows close clinical similarity with the features reported previously [16,17,20,21,22]. *METTL23* (MIM 615262) belongs to a small family of methyltransferases, having several potential functions including the regulation of chaperone proteins, protein folding, DNA repair, histone modification, splicing factor regulation and signal transduction [17]. To date, nine disease-causing variants in *METTL23* have been associated with ID (Supplementary Table S1). While this small single domain protein seems to exclude specific genotype–phenotype correlations, all AR or compound heterozygous variants reported so far in *METTL23* have been associated with ID with various degrees of facial dysmorphism [16,17,20,21]. This is also the case for the two patients presented here, who have several common features with previously reported cases including large eyes, short upturned nose, flat nasal bridge and thin lips. This observation may be helpful to improve diagnosis in the very diverse field of ID disorders as it confirms that *METTL23* may be a distinct clinical entity associating ID with specific facial dysmorphia. Functionally, due to its proximity to the SAH/SAM binding site, the variant may affect the ligand binding property of the protein and thereby impair its transcriptional regulation function [16,17].

Overall, the present study further supports previous findings that bi-allelic variants in *NDST1* and *METTL23* cause AR ID. Our findings expand the knowledge on genotype–phenotype correlations in ID related to *NDST1* and *METTL23* variants.

## Figures and Tables

**Table 1:** Clinical information of families A and B.

Clinical Findings	Family A		Family B		
Sex	M	F	F	F	F
Age (years)	12	10	24	12	10
Developmental delay	+	+	+	+	+
Intellectual disability	++	++	++	++	+
Microcephaly	-	-	-	-	-
Hypothyroidism	-	-	-	-	-
Speech development delay	+	+	-	-	-
Learning disability	+	+	+	+	+
Behavioral abnormalities	+	+	+	+	+
Dysmorphic features	-	-	+	+	-
Skeletal problem	-	-	-	-	-
Ophthalmic problem	-	-	-	-	-
Skin problem	-	-	-	-	-
Seizures	+	+	+	+	+
Social activity	+	+	+	+	-
Muscular dystrophy	-	-	-	-	-
Self biting	-	-	++	++	+

M, male; F, female; -, absence; +, mildly affected; ++, severely affected.

**Table 2:** Homozygous sequence variants identified in families A and B.

Family ID	Family A	Family B
Affected individuals	IV-1, IV-2	V-2, V-4, V-5
Transcript ID	NM_001543.5	NM_001206984.3
Gene name	*NDST1*	*METTL23*
MIM number	616116	615942
Chromosome position	Chr5:149922529	Chr17:74729285
Nucleotide change	c.1966G>A	c.310T>C
Protein change	p.Asp656Asn	p.Phe104Leu
SNP number	rs150320391	-
GnomAD frequency	0.000016	-
SIFT score	0.08/T	0/D
Polyphen2 score	0.992/D	0.891/PD
Mutation taster score	1/D	1/D
CADD score	27.4	31
PROVEAN score	−0.17/N	−4.67/D
ACMG classification	PM1	PM2

T, tolerated; N, neutral; D, damaging; PD, probably damaging.

## Supplementary Materials

The following are available online at https://www.mdpi.com/2073-4425/11/9/1021/s1, Table S1: Details of variants identified in *NDST1* and *METTL23*.
