# Supplementary material for: Two Cases of Recessive Intellectual Disability Caused by NDST1 and METTL23 Variants
Source: Genes (Basel). 2020 Aug 31;11(9):1021. doi: 10.3390/genes11091021 (PMC7563614; doi:10.3390/genes11091021)
Supplement: Supplementary file 1 [file genes-11-01021-s001.pdf]

Supplemental Table 1. **Details of variants identified in *NDST1* and *METTL23*.**

Gene	DNA Variation	Protein Variation	Variant Type	Reported phenotype	Reference[1]
NDST1	c.985C>T	p.Arg329Cys	Missense	Hypotonia & seizures	(Monies et al., 2017)
	deletion 70 kb	-	Deletion	Autism spectrum disorder	(Bitar et al., 2019)
	c.1114G>T	p.Ala372Ser	Missense	Developmental delay, ataxia, cranial nerve palsies & severe respiratory problems	(Armstrong et al., 2017)
	c.1360C>T	p.Arg454Cys	Missense	Developmental delay, coarse facies	(Gupta et al. 2019)
	1766A>G	p.Lys589Arg	Missense	Epilepsy, early-onset	(Demos et al., 2019)
	c.1831G>A	p.Gly611Ser	Missense	Intellectual disability	(Reuter et al., 2014)
	c.1918T>C	p.Phe640Leu	Missense	Intellectual disability	(Reuter et al., 2014)
	c.1926G>T	p.Glu642Asp	Missense	Intellectual disability	(Reuter et al., 2014)
	c.2126G>A	p.Arg709Gln	Missense	Intellectual disability	(Reuter et al., 2014)
	c.2207C>T	p.Ala736Val	Missense	Developmental delay, ataxia, cranial nerve palsies & severe respiratory problems	(Armstrong et al., 2017)
	c.2218G>A	p.Ala740Thr	Missense	Epilepsy, early-onset	(Demos et al., 2019)
	c.1966G>A	p.Asp656Asn	Missense	Intellectual disability	This study
METTL23	c.169_172delCACT	p.(His57Valfs*11)	Frameshift Deletion	Intellectual disability	(Reiff et al., 2014)
	c.176_177insG	p.Glu60Glyfs*11	Frameshift Insertion	Intellectual disability	(Smaili et al., 2020)
	c.204_206delGAA	p.Met68del	Inframe Deletion	Autism spectrum disorder	(Iossifov et al., 2014)
	c.237_241delAACAT	p.(Thr80Glyfs*20)	Frameshift Deletion	Intellectual disability	(Harripaul et al., 2018)
	c.282_286delAGATA	p.(Gln94Hisfs*6)	Frameshift Deletion	Intellectual disability	(Bernkopf et al., 2014)
	c.322+2T>C	-	Canonical-splice	Intellectual disability	(Almannai et al., 2020)
	c.397C>T	p.Gln133*	Nonsense	Intellectual disability	(Bernkopf et al., 2014)
	c.407+1G>C	-	Canonical-splice	Intellectual disability	(Almannai et al., 2020)
	c.449T>C	Met150Thr	Missense	Intellectual disability	(Almannai et al., 2020)
	c.470_471delTT	p.(Leu157Argfs*4)	Frameshift Deletion	Intellectual disability	(Almannai et al., 2020)
	c.310T>C	p.Phe104Leu	Missense	Intellectual disability	This Study

[1] References

Almannai, M.; Obaid, O.; Faqeih, E.; Alasmari, A.; Samman, M.M.; Pinz, H.; Braddock, S.R.; Alkuraya, F.S. Further delineation of METTL23-associated intellectual disability. American journal of medical genetics. Part A 2020, 182, 785-791, doi:10.1002/ajmg.a.61503.

Armstrong, L., Tarailo-Graovac, M., Sinclair, G., Seath, K.I., Wasserman, W.W., Ross, C.J., van Karnebeek, C.D., 2017. A girl with developmental delay, ataxia, cranial nerve palsies, severe respiratory problems in infancy-Expanding NDST1 syndrome. American journal of medical genetics. Part A 173(3), 712-715.

Bernkopf, M., Webersinke, G., Tongsook, C., Koyani, C.N., Rafiq, M.A., Ayaz, M., Muller, D., Enzinger, C., Aslam, M., Naeem, F., Schmidt, K., Gruber, K., Speicher, M.R., Malle, E., Macheroux, P., Ayub, M., Vincent, J.B., Windpassinger,

C., Duba, H.C., 2014. Disruption of the methyltransferase-like 23 gene METTL23 causes mild autosomal recessive intellectual disability. Human molecular genetics 23(15), 4015-4023.

Bitar, T., Hleihel, W., Marouillat, S., Vonwill, S., Vuillaume, M.L., Soufia, M., Vourc'h, P., Laumonnier, F., Andres, C.R., 2019. Identification of rare copy number variations reveals PJA2, APCS, SYNPO, and TAC1 as novel candidate genes in Autism Spectrum Disorders. Molecular genetics & genomic medicine 7(8), e786.

Demos, M., Guella, I., DeGuzman, C., McKenzie, M.B., Buerki, S.E., Evans, D.M., Toyota, E.B., Boelman, C., Huh, L.L., Datta, A., Michoulas, A., Selby, K., Bjornson, B.H., Horvath, G., Lopez-Rangel, E., van Karnebeek, C.D.M., Salvarinova, R., Slade, E., Eydoux, P., Adam, S., Van Allen, M.I., Nelson, T.N., Bolbocean, C., Connolly, M.B., Farrer, M.J., 2019. Diagnostic Yield and Treatment Impact of Targeted Exome Sequencing in Early-Onset Epilepsy. Frontiers in neurology 10, 434.

Harripaul, R., Vasli, N., Mikhailov, A., Rafiq, M.A., Mittal, K., Windpassinger, C., Sheikh, T.I., Noor, A., Mahmood, H., Downey, S., Johnson, M., Vleuten, K., Bell, L., Ilyas, M., Khan, F.S., Khan, V., Moradi, M., Ayaz, M., Naeem, F., Heidari, A., Ahmed, I., Ghadami, S., Agha, Z., Zeinali, S., Qamar, R., Mozhdehipanah, H., John, P., Mir, A., Ansar, M., French, L., Ayub, M., Vincent, J.B., 2018. Mapping autosomal recessive intellectual disability: combined microarray and exome sequencing identifies 26 novel candidate genes in 192 consanguineous families. Molecular psychiatry 23(4), 973-984.

Iossifov, I., O'Roak, B.J., Sanders, S.J., Ronemus, M., Krumm, N., Levy, D., Stessman, H.A., Witherspoon, K.T., Vives, L., Patterson, K.E., Smith, J.D., Paeper, B., Nickerson, D.A., Dea, J., Dong, S., Gonzalez, L.E., Mandell, J.D., Mane, S.M., Murtha, M.T., Sullivan, C.A., Walker, M.F., Waqar, Z., Wei, L., Willsey, A.J., Yamrom, B., Lee, Y.H., Grabowska, E., Dalkic, E., Wang, Z., Marks, S., Andrews, P., Leotta, A., Kendall, J., Hakker, I., Rosenbaum, J., Ma, B., Rodgers, L., Troge, J., Narzisi, G., Yoon, S., Schatz, M.C., Ye, K., McCombie, W.R., Shendure, J., Eichler, E.E., State, M.W., Wigler, M., 2014. The contribution of de novo coding mutations to autism spectrum disorder. Nature 515(7526), 216-221.

Monies, D., Abouelhoda, M., AlSayed, M., Alhassnan, Z., Alotaibi, M., Kayyali, H., Al-Owain, M., Shah, A., Rahbeeni, Z., Al-Muhaizea, M.A., Alzaidan, H.I., Cupler, E., Bohlega, S., Faqeih, E., Faden, M., Alyounes, B., Jaroudi, D., Goljan, E., Elbardisy, H., Akilan, A., Albar, R., Aldhalaan, H., Gulab, S., Chedrawi, A., Al Saud, B.K., Kurdi, W., Makhseed, N., Alqasim, T., El Khashab, H.Y., Al-Mousa, H., Alhashem, A., Kanaan, I., Algoufi, T., Alsaleem, K., Basha, T.A., Al-Murshedi, F., Khan, S., Al-Kindy, A., Alnemer, M., Al-Hajjar, S., Alyamani, S., Aldhekri, H., Al-Mehaidib, A., Arnaout, R., Dabbagh, O., Shagrani, M., Broering, D., Tulbah, M., Alqassmi, A., Almugbel, M., AlQuaiz, M., Alsaman, A., Al-Thihli, K., Sulaiman, R.A., Al-Dekhail, W., Alsaegh, A., Bashiri, F.A., Qari, A., Alhomadi, S., Alkuraya, H., Alsebayel, M., Hamad, M.H., Szonyi, L., Abaalkhail, F., Al-Mayouf, S.M., Almojalli, H., Alqadi, K.S., Elsiesy, H., Shuaib, T.M., Seidahmed, M.Z., Abosoudah, I., Akleh, H., AlGhonaium, A., Alkharfy, T.M., Al Mutairi, F., Eyaid, W., Alshanbary, A., Sheikh, F.R., Alsohaibani, F.I., Alsonbul, A., Al Tala, S., Balkhy, S., Bassiouni, R., Alenizi, A.S., Hussein, M.H., Hassan, S., Khalil, M., Tabarki, B., Alshahwan, S., Oshi, A., Sabr, Y., Alsaadoun, S., Salih, M.A., Mohamed, S., Sultana, H., Tamim, A., El-Haj, M., Alshahrani, S., Bubshait, D.K., Alfadhel, M., Faquih, T., El-Kalioby, M., Subhani, S., Shah, Z., Moghrabi, N., Meyer, B.F., Alkuraya, F.S., 2017. The landscape of genetic diseases in Saudi Arabia based on the first 1000 diagnostic panels and exomes. Human genetics 136(8), 921-939.

Reiff, R.E., Ali, B.R., Baron, B., Yu, T.W., Ben-Salem, S., Coulter, M.E., Schubert, C.R., Hill, R.S., Akawi, N.A., Al-Younes, B., Kaya, N., Evrony, G.D., Al-Saffar, M., Felie, J.M., Partlow, J.N., Sunu, C.M., Schembri-Wismayer, P.,

Alkuraya, F.S., Meyer, B.F., Walsh, C.A., Al-Gazali, L., Mochida, G.H., 2014. METTL23, a transcriptional partner of GABPA, is essential for human cognition. Human molecular genetics 23(13), 3456-3466.

Reuter, M.S., Musante, L., Hu, H., Diederich, S., Sticht, H., Ekici, A.B., Uebe, S., Wienker, T.F., Bartsch, O., Zechner, U., Oppitz, C., Keleman, K., Jamra, R.A., Najmabadi, H., Schweiger, S., Reis, A., Kahrizi, K., 2014. NDST1 missense mutations in autosomal recessive intellectual disability. American journal of medical genetics. Part A 164A(11), 2753-2763.

Smaili, W.; Elalaoui, S.C.; Zrhidri, A.; Raymond, L.; Egea, G.; Taoudi, M.; Mouatassim, S.E.L.; Sefiani, A.; Lyahyai, J. Exome sequencing revealed a novel homozygous METTL23 gene mutation leading to familial mild intellectual disability with dysmorphic features. European journal of medical genetics 2020, 63, 103951, doi:10.1016/j.ejmg.2020.103951.

© 2020 by the authors. Submitted for possible open access publication under the terms and conditions of the Creative Commons Attribution (CC BY) license (http://creativecommons.org/licenses/by/4.0/).
